# On the Optimal Field Sensing in Near-Field Characterization

**DOI:** 10.3390/s21134460

**Published:** 2021-06-29

**Authors:** Amedeo Capozzoli, Claudio Curcio, Angelo Liseno

**Affiliations:** Dipartimento di Ingegneria Elettrica e delle Tecnologie dell’Informazione, Universitá di Napoli Federico II, via Claudio 21, 80125 Napoli, Italy; clcurcio@unina.it (C.C.); angelo.liseno@unina.it (A.L.)

**Keywords:** near-field/far-field transformations, source/scatterer characterization, singular value optimization, singular value decomposition, gaussian quadrature, optimality

## Abstract

We deal with the problem of characterizing a source or scatterer from electromagnetic radiated or scattered field measurements. The problem refers to the amplitude and phase measurements which has applications also to interferometric approaches at optical frequencies. From low frequencies (microwaves) to high frequencies or optics, application examples are near-field/far-field transformations, object restoration from measurements within a pupil, near-field THz imaging, optical coherence tomography and ptychography. When analyzing the transmitting-sensing system, we can define “optimal virtual” sensors by using the Singular Value Decomposition (SVD) approach which has been, since long time, recognized as the “optimal” tool to manage linear algebraic problems. The problem however emerges of discretizing the relevant singular functions, thus defining the field sampling. To this end, we have recently developed an approach based on the Singular Value Optimization (SVO) technique. To make the “virtual” sensors physically realizable, in this paper, two approaches are considered: casting the “virtual” field sensors into arrays reaching the same performance of the “virtual” ones; operating a segmentation of the receiver. Concerning the array case, two ways are followed: synthesize the array by a generalized Gaussian quadrature discretizing the linear reception functionals and use elementary sensors according to SVO. We show that SVO is “optimal” in the sense that it leads to the use of elementary, non-uniformly located field sensors having the same performance of the “virtual” sensors and that generalized Gaussian quadrature has essentially the same performance.

## 1. Introduction

Near-field source characterization consists of reconstructing the radiating features of a transmitter by measuring its radiated field in the near-zone using field sensors [1,2,3,4]. Such a problem arises in different fields of applied electromagnetics as near-field antenna characterization [1,2,3] and near-field scanning of electromagnetic emissions [5]. We face here amplitude and phase sampling which has applications also to interferometric approaches at optical frequencies. Relevant to the high-frequency and optical regimes, we mention the characterization of thermal fields [6] and of laser beams [7], near-field THz imaging [8], optical coherence tomography [9] and ptychography [10] as application examples.

When analyzing a transmitting-sensing system, proper basis functions can be introduced to properly represent the field on the transmitter and on the sensor ends. Furthermore, “virtual” transmitters and sensors, that take into account the geometry of the link, can be defined, regardless to being physically implementable or not. For a fixed transmitter, these are “optimal” if derived by the Singular Value Decomposition (SVD) approach which is recognized as the “optimal” tool to manage linear algebraic problems. This point has been recently addressed in [11]. Indeed, under general hypotheses on the noise and weak geometrical assumptions, the SVD assigns a precise meaning to the concept of Degrees of Freedom (DoFs) [12,13]. It identifies the actual space dimensions when resorting to the singular system corresponding to the most significant singular values. The singular system defines the space of “optimal” transmitters and that of “optimal” receivers [12,13]. Therefore, the “virtual” sensors defined by the SVD are “optimal” since they are the minimum number of sensors capable to extract all the information of the field radiated by the transmitter belonging to a specified class of transmitters on the measurement area and for the considered geometry of the link. They are “virtual” because, as will be remarked below, the most convenient representation is by arrays or segmented sensors.

Besides the theoretical interest of properly identifying the DoFs of the radiated field on a region of space, investigations on the problem at hand have been repeatedly proposed and used, in the field of imaging optics, to attempt providing an answer to issues related to the resolution of an image [14], to restoring objects beyond the diffraction limit [15] and to evaluating the information content of optical wave fields [11,13].

Generally speaking, the “optimal virtual” sensors defined by the singular functions of the SVD have the entire sensing area as support. Consequently, they overlap. Due to the overlap, the acquisition of the field radiated by the transmitter must be performed by *N* temporarily subsequent measurements with *N* different sensors, where *N* is the number of “optimal” sensors. Performing *N* subsequent measures with *N* different sensors can be impractical in terms of measurement duration. Moreover, the problem arises of how synthesizing and implementing the “optimal virtual” sensors starting from the singular functions defined by the SVD.

To make the “virtual” sensors physically realizable avoiding the overlap, two approaches are here conceived.

1. The “virtual” field sensors can be cast into arrays, so that each “virtual” sensor corresponds to an array; each array should be conceived so that it reaches the same performance of the “virtual” field sensor to which it corresponds. The array is the natural representation of a “virtual” receiver following the discrete representation of a continuous measurement functional by quadrature or an equivalent process, where the continuous functional is the mathematical representation of the “virtual” sensors.
  Casting the “virtual” field sensors into arrays makes their realization practical since the arrays can use elementary sensors which “sample” the radiated field on the sensing area. The complexity of the synthesis of the individual extended sensor is overturned to the way the differently collected data from the different sensors are combined. Furthermore, the arrays can be conceived to have all the same number of elements sharing the same positions and have the advantage that their synthesis amounts to the determination of proper weights for the acquired field samples enabling the discretization of the “virtual” sensors. Finally, synthesizing the arrays can be carried out as illustrated below.
2. It is possible to operate a segmentation of the receiver so to make the sensors spatially disjoint. The segmentation solves the overlap issue. However, differently from before, depending on how it is performed, the segmentation does not exploit elementary sensors so that the problem of properly synthesizing the different segments remains. Throughout the paper, we will consider the solution at the foregoing point.

Concerning the array case, two ways can be followed:

1.A Use proper “optimized” generalized Gaussian quadrature rules [16] to discretize linear reception functionals.
1.B Use elementary sensors as in case 1.A, but now following the definition of “optimized” sensor locations [1,2,17].

Turning apertures into arrays is a practice, but defining the array to have the same performance of the un-discretized setup is an overlooked issue.

After the first goal of the paper of defining the “virtual” sensors, our further goals are then:

- introduce approaches 1.A and #2 which are new ideas of this paper;
- show for the very first time that approach #1.B, namely the sampling approach provided by the Singular Value Optimization (SVO), which has been already introduced by the Authors, is “optimal” in the sense that it leads to the use of elementary, non-uniformly located field sensors having the same performance of the impractical “virtual” ones.

In this sense, Gaussian quadrature represents an alternative to SVO.

Several contributions to SVO have appeared throughout the literature with a target different from the one pursued in the present paper. In [18,19], SVO has been used for plane-polar near-field acquisitions; in [20,21], it has been exploited for very-near-field measurements performed with dielectric probes; in [2], a multi-frequency extension has been given; in [17], it has been applied to a helicoidal cylindrical scanning; in [3], a criterion to determine the size of the portion of the measurement plane and the “quasi-raster” scanning have been introduced; in [22], SVO has been extended to the case of incoherent sources; in [23,24], it has been extended to inverse scattering, also with multi-resolution purposes; in [25], the use of gradient information in the optimization of the sample locations has been introduced; in [26,27], SVO has been used in connection to the design and construction of an innovative scanner controller; finally, in [28,29], extensions to the spherical and cylindrical scanning geometries have been provided.

We note that other techniques have been already proposed for non-uniform field sampling, especially at optical frequencies, see [30,31,32,33]. SVO has been already compared to other sampling techniques in [1] showing superior performance.

We stress that the behavior of the system composed of the transmitter and the sensor can be described in a synthetic and abstract way by means of linear functionals, extending to the general case the standard concept of effective length.

**Figure 1:**
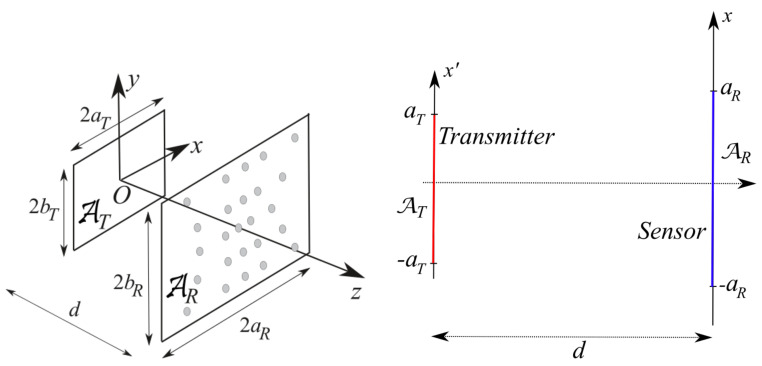
Left: geometry of the 3D problem. Right: geometry of the 2D problem.

For the sake of simplicity, but without loss of generality, the geometry considered in this paper has planar rectangular and parallel transmitting and sensing domains (Figure 1) [34].

All the inversions throughout the paper are performed by the Truncated SVD (TSVD) since the TSVD of the un-discretized radiation operator has optimal performance in case of additive, uncorrelated, white Gaussian noise [35].

The validity of SVO has been extensively experimentally verified in various scanning geometries [2,3,17,18,19,20,21,22,23,24,25,26,27,28,29]. The purpose of this paper is showing the “optimality” of SVO by formulating the problem in a rigorous mathematical setting and also proving that other possible solutions, related to the representation of the measurement functionals through generalized quadrature, lead essentially to the same results.

The paper is organized as follows. In Section 2, the sensing process is regarded as a scattering process and the effective length for aperture sensors is drawn as the result of a rigorous scattering approach of the receiving process. In Section 3, a simple, scalar 2D problem is considered and the “optimal virtual” sensors defined. Section 4 introduces the different possibilities to practically implement the “optimal virtual” sensors. Section 5 collects the performance analysis and the discussion. Finally, Section 6 gathers the conclusions.

## 2. Aperture Modelling of Transmitter and Sensor

In this section, we briefly recall the aperture modelling of the transmitter. Furthermore, the aperture modelling problem of the sensor is faced in its very nature, namely, as a scattering problem and the concept of effective length for aperture sensors is rigorously drawn as a new result by using mathematical theorems of functional analysis [36].

The modelling of the transmitter and the sensor as apertures is necessary to rigorously define the following vector subspaces:

1. subspace to which all the possible fields impinging on the sensors essentially belong;
2. subspace to which all the possible fields impinging on the sensor that are actually receivable belongs to, thanks to the evaluation of the sensed signal as a scalar product.

### 2.1. Modelling of the Transmitter

The transmitter is assumed to be modelled with a radiating aperture whose domain $A_{T}$ is $2a_{T}\times 2b_{T}$ sized and the transmitter is located on the z=0 plane (see Figure 1—left). For the sake of simplicity, but without any loss in generality, we consider the case of a *y*-polarized aperture field ${\underline{E}}_{a}=E_{a}{\hat{ı}}_{y}$. For the case of parallel aperture and sensing domains, the plane-wave expansion shows that the fields are fully determined by the transverse spectrum [37]. The two Cartesian transverse *x*- and *y*-components propagate from the radiating aperture to the sensing plane independently each other. Accordingly, a *y*-polarized aperture field leads to dealing with a scalar problem. The aperture field is assumed to be vanishing outside $A_{T}$.

### 2.2. Modelling of the Sensor

**Figure 2:**
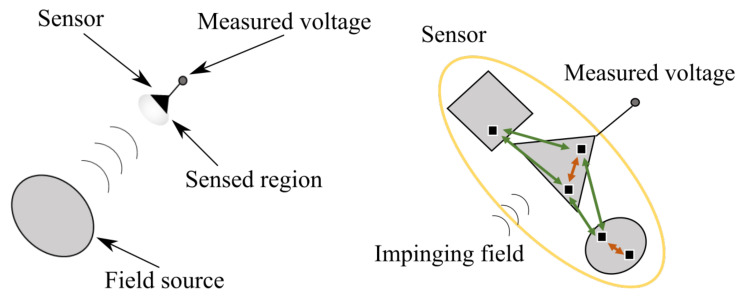
The sensing process as a scattering one.

We now describe the sensing process as a scattering process, see Figure 2—left, and generalize the concept of effective length.

#### 2.2.1. The Sensing Process as a Scattering Process

The source in Figure 2—left radiates the impinging field over the sensed region, that is, the region physically occupied by the sensor. The sensor extracts information from the impinging field in the sensed region through the total field, namely, the sum of the impinging field and the field scattered by the different parts of the sensor (Figure 2—right). Finally, the sensor makes a signal *V* available which then depends on the total field at the probe terminals. The total field and, thus, *V* can be determined only after solving a full scattering problem. *V* is then determined by all the portions of the probe that interact with the impinging field and that electromagnetically interact each other giving rise to mutual interactions. Notice that the probe is not forced to fill, geometrically, a connected domain and can be made of separated parts that interact each other when generating the scattered (and the total) field. Typically, these parts are thought of as disconnected from the main body of the probe and dealt with as parasite elements. Accordingly, the sensor is the whole system, and all the mutual interactions between its portions (red and green parts of Figure 2—right) that generate the scattered (and total) field have the same conceptual nature [38].

#### 2.2.2. The Effective Length and the Aperture Modelling of the Receiver

As long as the probing sensor is made of linear materials and linear components, the relationship between the impinging field in the sensed region and the sensed signal is represented by a linear functional, say M

(1) $$M\;\;|\;\;{\underline{E}}_{i}\;\;\to \;\;M\left({\underline{E}}_{i}\right)=\langle {\underline{E}}_{i},\underline{M}\rangle =V,$$

where ${\underline{E}}_{i}$ contains the relevant vector components of the field incident all over the sensed region $A_{R}$ (see Figure 1—left) as the function of (x,y), M returns the signal from the impinging field, 〈·,·〉 stands for the duality and $\underline{M}$ is the vector function representing the functional M in the dual space of the fields.

The aperture modelling of the sensor assumes that the only portion of the impinging field relevant to the scattering process amounts to that on the aperture. Accordingly, after the Ritz Theorem [36] and for a planar sensing aperture, M can be expressed as a scalar product

(2) $$M|{\underline{E}}_{i}\;\;\to \;\;{\;\int \int \;}_{AR}{\underline{E}}_{i}\left(x,y\right)\cdot {\underline{m}}^{*}\left(x,y\right)dxdy=V,$$

where $\underline{m}\left(x,y\right)$ represents the sensing function and generalizes the concept of effective length. According to (2), for a planar sensor, it is sufficient to know the impinging field only on the probe aperture $A_{R}$. Indeed, the impinging field on the sensing aperture is all that is needed to work out the scattering process.

The expression of M in Equations (1) and (2) are more familiar than what appears at the first sight. Indeed, let us consider the case when ${\underline{E}}_{i}$ can be approximated by a plane wave in the whole sensed region, namely ${\underline{E}}_{i}={\underline{E}}_{i_{0}}\exp\left(-j\beta \hat{k}\cdot \underline{r}\right)$, where $\underline{r}$ is the position vector and $\hat{k}$ is the unit propagation vector. In this case, and on substituting the plane wave expression (2), *V* turns into

(3) $$V={\underline{E}}_{i_{0}}\cdot {\underline{l}}_{e}\left(\hat{k}\right)$$

which expresses the well-known concept of effective length ${\underline{l}}_{e}$ of the sensor.

Finally, the integral in Equation (2) can be extended to $R^{2}$ by assuming $\underline{m}$ vanishing outside $A_{R}$, namely

(4) $$M|{\underline{E}}_{i}\;\;\to \;\;{\;\int \int \;}_{R^{2}}{\underline{E}}_{i}\left(x,y\right)\cdot {\underline{m}}^{*}\left(x,y\right)dxdy=V.$$

Note that the ${\underline{m}}^{*}\left(x,y\right)$ appearing in Equation (4) rigorously accounts for all the scattering mechanisms and so it accounts for a full-wave description of the sensor.

The scalar product (2) has a spectral domain expression, according to the Parseval–Plancherel equality, as

(5) $$M|{\underline{E}}_{i}\to \frac{1}{{\left(2\pi \right)}^{2}}{\;\int \int \;}_{R^{2}}{\hat{\underline{E}}}_{i}\left(k_{x},k_{y}\right)\cdot {\hat{\underline{m}}}^{*}\left(k_{x},k_{y}\right)dk_{x}dk_{y}.$$

As long as the sensor is outside the reactive region of the transmitter, being ${\underline{E}}_{i}$ vanishing outside the visible domain $V=\{\left(k_{x},k_{y}\right)|k_{x}^{2}+k_{y}^{2}\leq \beta^{2}\}$, where β=2π/λ, λ is the wavelength and $k_{x}$ and $k_{y}$ are the spectral variables, the field $\hat{m}$ can be assumed zero, correspondingly. Therefore, Equation (5) can be expressed as

(6) $$M|{\underline{E}}_{i}\to \frac{1}{{\left(2\pi \right)}^{2}}{\;\int \int \;}_{V}{\hat{\underline{E}}}_{i}\left(k_{x},k_{y}\right)\cdot {\hat{\underline{m}}}^{*}\left(k_{x},k_{y}\right)dk_{x}dk_{y}.$$

#### 2.2.3. The space of Sensing Functions: The Scalar Case

By considering a scalar case with a scalar sensing function, Equations (2) and (6) become

(7) $$M|E_{i}\to {\;\int \int \;}_{AR}E_{i}\left(x,y\right)\cdot m^{*}\left(x,y\right)dxdy=V$$

and

(8) $$M|E_{i}\to \frac{1}{{\left(2\pi \right)}^{2}}{\;\int \int \;}_{V}{\hat{E}}_{i}\left(k_{x},k_{y}\right)\cdot {\hat{m}}^{*}\left(k_{x},k_{y}\right)dk_{x}dk_{y},$$

respectively.

The scalar field *m* then belongs to the subspace of functions with bounded support $A_{R}$ whose Fourier transform has essentially bounded support V. Accordingly, *m* belongs to the subspace generated by the Prolate Spheroidal Wave Functions (PSWFs) [1,2,3,39,40,41,42] associated to the sensing aperture.

## 3. “Optimal Virtual” Sensors

Following the result of the foregoing Section, we now introduce the “optimal virtual” sensors along with the expressions of their own sensing functions. For the reader’s convenience, the modelling for the transmitting and sensing apertures is worked out for the scalar 2D geometry in Figure 1—right. The modelling for the full 3D case can be obtained using function factorization along *x* and *y*. A full 3D example will be anyhow provided in Section 5.5.

Figure 1—right shows the case of planar and parallel domains, centered one other.

For the transmitting case, we assume that the aperture field $E_{a}\left(x^{\prime }\right)$ is linearly polarized along the *y*-axis. Then, the only (*y*) component of the aperture field $E_{a}\left(x^{\prime }\right)$ is represented by $K_{T}=\left\lfloor 2c_{T}/\pi \right\rceil$ PSWFs [1,2,3,39,40,41,42], where ⌊ξ⌉ is the nearest integer not smaller than ξ, and $c_{T}=a_{T}\beta$ is the space-bandwidth product, namely

(9) $$E_{a}\left(x^{\prime }\right)=\sum_{k=1}^{K_{T}}e_{k}\Phi_{k}\left[c_{T};x^{\prime }\right],$$

where $\Phi_{k}\left[\gamma ;x^{\prime }\right]$ is the *k*-th PSWF with space-bandwidth product γ and the $e_{k}$’s are the expansion coefficients.

For the sensing case, considering the obvious scalar, *y* polarized sensing function $\underline{m}\left(x\right)=m\left(x\right){ı}_{y}$, then m(x) is expressed as

(10) $$m\left(x\right)=\sum_{l=1}^{L_{R}}\mu_{l}\Phi_{l}\left[c_{R};x\right],$$

where $L_{R}=\left\lfloor 2c_{R}/\pi \right\rceil$, $c_{R}=a_{R}\beta$ and the $\mu_{l}$’s are expansion coefficients.

According to Equation (9), the field impinging onto the sensed region can be expressed as

(11) $$E_{i}\left(x\right)=A\left(E_{a}\right)\left(x\right)=\sum_{k=1}^{K_{T}}e_{k}A\left[\Phi_{k}\left[c_{T};x^{\prime }\right]\right]\left(x\right),$$

where A is the radiation operator linking the field on z=0 to that on z=d.

Care should be provided to the case when the apertures reach dimensions comparable or smaller than the wavelength which leads to small space-bandwidth products. In this case, it would be necessary to transiting to elementary transmitter and sensor representations.

According to Equations (7) and (10) and using Equation (11), the signal *V* is

(12) $$V=\langle A\left(E_{a}\right)\left(x\right),m\left(x\right)\rangle =\sum_{k=1}^{K_{T}}\sum_{l=1}^{L_{R}}e_{k}m_{l}A_{kl},$$

where $A_{kl}=\langle A\left[\Phi_{k}\left[c_{T};x^{\prime }\right]\right]\left(x\right),\Phi_{l}\left[c_{R};x\right]\rangle$.

In Equation (12), the sensed signal depends on the transmitter by the $e_{k}$’s and on the sensor by the $\mu_{l}$’s. Furthermore, the $A_{kl}$’s define the link matrix $\underline{\underline{A}}$ providing the connection between the *k*-th transmitting and the *l*-th sensing PSWFs.

For fixed values of $a_{T}$, $a_{R}$ and *d*, the “optimal virtual” sensors can be defined by the SVD of $\underline{\underline{A}}$ which factorizes $\underline{\underline{A}}$ as

(13) $$\underline{\underline{A}}=\underset{K_{T}\times K_{T}}{\underset{︸}{\underline{\underline{U}}}}\;\underset{K_{T}\times L_{R}}{\underset{︸}{\underline{\underline{\Sigma }}}}\;\underset{L_{R}\times L_{R}}{\underset{︸}{{\underline{\underline{V}}}^{†}}},$$

where the diagonal matrix $\underline{\underline{\Sigma }}$ contains the singular values $\sigma_{n}$ of $\underline{\underline{A}}$ and ${}^{†}$ denotes conjugate transposition. The rank of $\underline{\underline{A}}$ is limited by the minimum between the number of transmitting PSWFs $K_{T}$ and the number of sensing PSWFs $L_{R}$. The SVD in (13) defines $N_{opt}$ “virtual” sensors $m_{n}\left(x\right)$ corresponding to the most significant $\sigma_{n}$’s as

(14) $$m_{n}\left(x\right)=\sum_{l=1}^{L_{R}}v_{ln}\Phi_{l}\left[c_{R};x\right],\;\;\;n=1,\ldots ,N_{opt}$$

where $v_{ln}$ is the *l*-th component of the *n*-th singular vector ${\underline{v}}_{n}$ which corresponds to the *n*-th column of $\underline{\underline{V}}$.

For a general system, the link is not able to capture all the DoFs of the transmitter if $N_{opt}<K_{T}$. As long as the distance between the transmitter and the sensor grows, the energy of the radiated PSWFs spreads and larger sensors are needed to recover all the radiated DoFs. This point will be discussed and numerically illustrated in Section 5.1.

We remark that the field radiated by the aperture is composed by the two contributions due to the visible and the invisible PSWFs. In the Very Near Field (VNF), or reactive, region of the aperture, both the contributions are significant. Opposite to that, few wavelengths away from the aperture, invisible components of the radiated field are negligible even if still in the near-field of the aperture. Throughout the paper, we consider sensors located outside the reactive region. Accordingly, the PSWFs are employed since they represent a basis for the visible part, namely, the only relevant part of aperture fields. Similar considerations apply to the sensing aperture.

By the PSWFs, the radiation operator and the sensing process are discretized and the link matrix $\underline{\underline{A}}$ defined. The “optimal virtual” receivers are then defined according to the SVD of the link matrix, as expressed by Equation (14).

## 4. Practical Realization of “Optimal Virtual” Sensors

**Figure 3:**
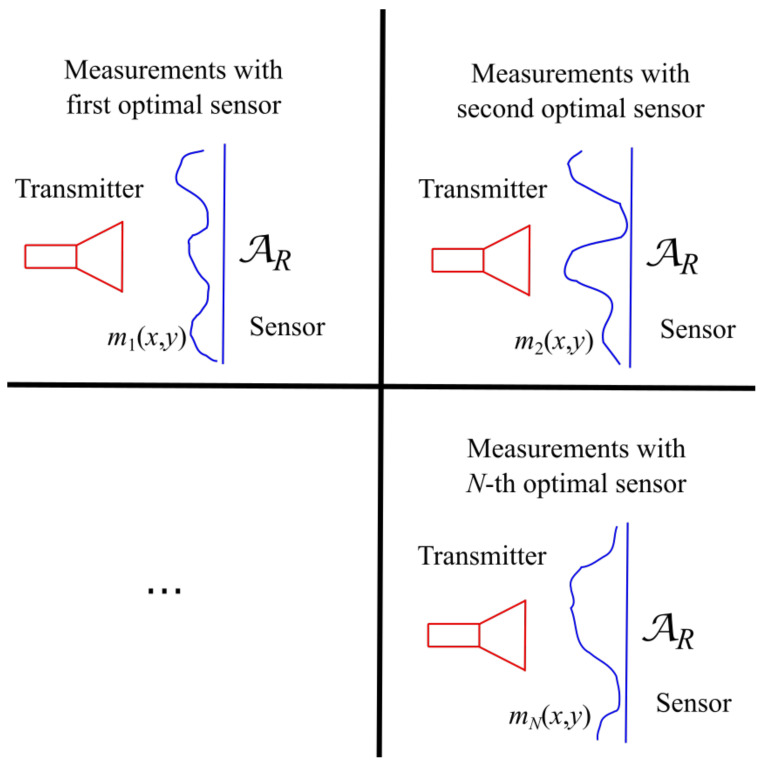
Subsequent use of the “optimal virtual” sensors to measure the transmitted field according to Equations (7) and (8).

The geometrical extent of each “optimal virtual” sensors, in general, embraces the whole aperture $A_{R}$ causing an unavoidable overlap issue among the various sensors. Accordingly, the “optimal virtual” sensors must be employed one after the other as in Figure 3 and the problem amounts to practically synthesize the receivers according to their own sensing functions $m_{n}\left(x,y\right)$ [34].

An alternative is represented by arrays of elementary sensors whose definition requires a discretization of the involved apertures.

Arrays are an appealing solution since:

1. they suggest the way on how synthesizing the sensors, since only the Network Weights (NWs) need to be determined, while the elementary composing elements do not need a substancial design at this stage;
2. they potentially solve the overlap issue, provided that the arrays of elementary sensors realize different receivers sharing the element positions, but realizes different sensors according to different sets of NWs.

The arrays can be realized by using “point-like” elements or extended elements. The NW can be physical or numerical.

We consider first point-like elements and then sketch on extended ones.

### 4.1. Elementary Sensors Using Generalized Gaussian Quadrature

The use of the sensing arrays corresponds to the discretization of the reception integrals (7) which is possible thanks to generalized Gaussian quadrature formulas.

**Figure 4:**
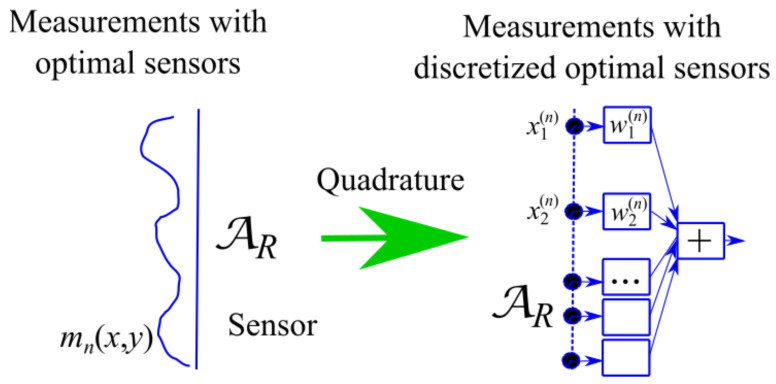
Illustrating the representation of the “optimal virtual” sensors by arrays thanks to the use of generalized Gaussian quadrature rules.

Applying generalized Gaussian quadrature means setting the following approximation

(15) $$\int_{-a_{R}}^{a_{R}}E_{i}\left(x\right)m_{n}^{*}\left(x\right)dx≃\sum_{s=1}^{N}w_{s}^{\left(n\right)}E_{i}\left(x_{s}^{\left(n\right)}\right),\;\;\forall E_{i}\in E_{i},n=1,\ldots ,N_{opt}.$$

where $E_{i}$ is the space spanned by the $K_{T}$ functions $A\left[\Phi_{k}\left[c_{T};x^{\prime }\right]\right]\left(x\right)$, see Equation (11). In (15), the number *N* of quadrature nodes and the nodes $x_{s}^{\left(n\right)}$’s and weights $w_{s}^{\left(n\right)}$’s define the array element number and (non-uniform, in general) locations, and the NWs, respectively, see Figure 4. They must guarantee a good approximation in (15) for all $E_{i}$ in $E_{i}$ for each fixed “optimal virtual” sensor. Obviously, quadrature involves an implicit sampling of the impinging field tuned to the problem aim.

**Figure 5:**
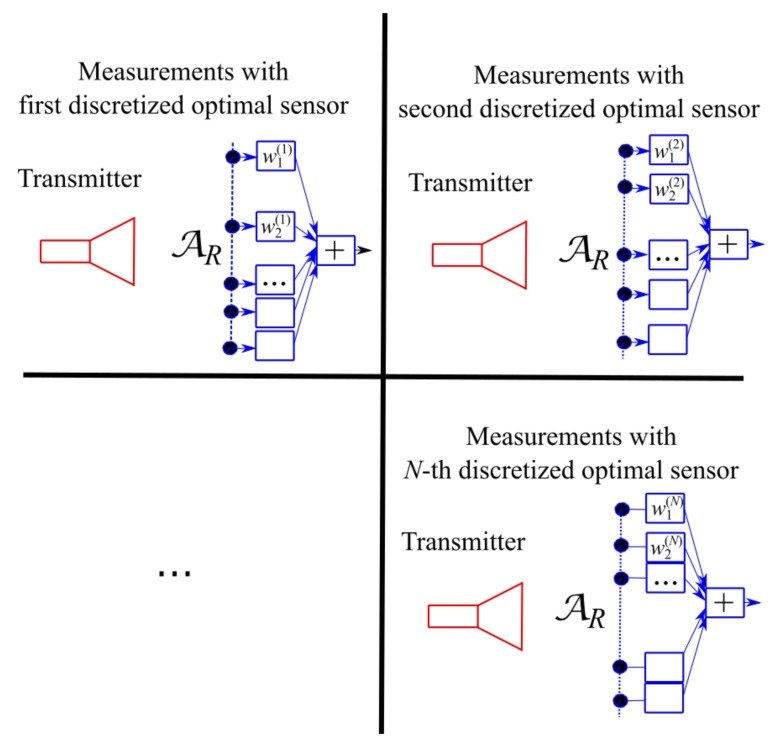
Subsequent use of the discretized “optimal virtual” sensors to measure the transmitted field.

Nevertheless, the application of Gaussian quadrature for each “optimal virtual” sensor separately leads to array element positions which can change across the sensors, see Figure 5. If the element positions must keep the same across the different “optimal virtual” sensors because we want to deal with a unique set of array elements, then we must enforce a constraint.

To achieve this purpose, a generalized Gaussian quadrature technique is introduced determining weights to approximate at the best the signals sensed for each possible $E_{i}$ in $E_{i}$ and for all the “optimal virtual” sensors as well as a common node grid for all the “optimal virtual” sensors. In other words, the integrals (7) are approximated as

(16) $$\int_{-a_{R}}^{a_{R}}E_{i}\left(x\right)m_{n}^{*}\left(x\right)dx≃\sum_{s=1}^{N}w_{s}^{\left(n\right)}E_{i}\left(x_{s}\right),\;\;\forall E_{i}\in E_{i},\;\;\;\forall m_{n},\;n=1,\ldots ,N_{opt}.$$

This task is simplified by the fact that both the impinging fields and the “optimal” reception functions belong to a finite dimensional space.

**Figure 6:**
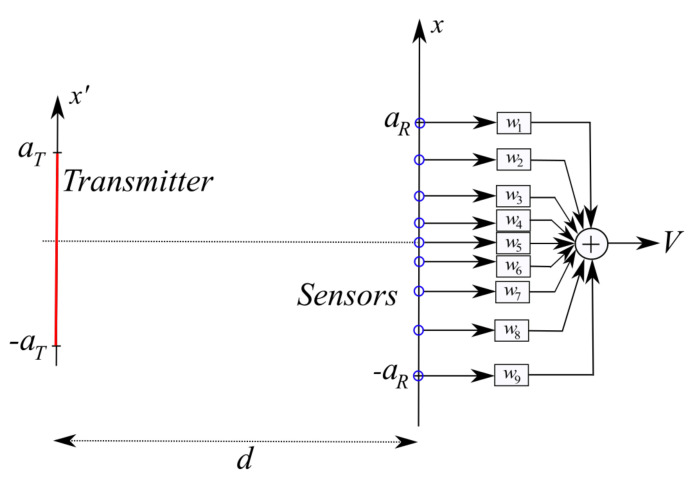
Illustrating the point-like elements array geometry with weights.

In other words, we consider the solution illustrated in Figure 6 consisting of arrays made of elementary sensors sharing number and positions of their elements. The arrays differentiate by only the weights ${\underline{w}}^{\left(n\right)}$, $n=1,\ldots ,N_{opt}$.

According to Equation (16), the array parameters are selected so that:

$$V_{nk}=\int_{-a_{R}}^{a_{R}}m_{n}^{*}\left(x\right)A\left[\Phi_{k}\left[c_{T};x^{\prime }\right]\right]\left(x\right)dx≃\sum_{s=1}^{N}w_{s}^{\left(n\right)}A\left[\Phi_{k}\left[c_{T};x^{\prime }\right]\right]\left(x_{s}\right),\;\;\;\;\;\;\;\;\;\;\;\;\;\;\;\;\;\;\;\;$$

(17) $$n=1,\ldots ,N_{opt},\;\;k=1,\ldots ,K_{T}.$$

Therefore, the synthesis of the positions of the elementary sensors as well as of their weights is worked out by solving a set of non-linear equations. The $V_{nk}$’s in Equation (17) are the signals sensed by the *n*-th “optimal virtual” sensor when the impinging field is produced by a transmitter with aperture field equal to the *k*-th radiating PSWFs.

### 4.2. SVO

Another possibility to synthesize the sensors while solving the overlap issue is using SVO which leads to represent the “optimal” sensing probes using a single array of point-like elements with element locations shared among the sensing aperture. Differently from Gaussian quadrature, SVO maximizes the amount of information on the source collected by the field samples $V_{s}$ acquired over $A_{R}$. We stress that SVO optimizes the Singular Value Behavior (SVB) of $\underline{\underline{A}}$ and that different metric to evaluate the degree of conditioning of the problem have been proposed, including the use of Shannon number, mutual information and Fisher information, see [43]. We mention that an alternative metrics can be obtained by resorting to the Hilbert–Schmidt norm of $\underline{\underline{A}}$, as suggested in [11].

Notice that SVO hides the explicit use of weights for the signals acquired by the individual elementary sensors. The absence of a mechanism similar to the quadrature weights is only apparent since they are actually introduced when the signals acquired by the individual sensors are processed.

A recall of the salient features of SVO is now in order.

#### SVO in Electromagnetics

In many electromagnetic contexts, the model describing the system is provided by a linear operator T mapping the input *a* into the output *b* and depending on *P* parameters $\underline{p}=\left(p_{1},\ldots ,p_{P}\right)$, namely

(18) $$T(a,\underline{p})=b.$$

Typically, *b* is available in terms of its discrete counterpart, expressed by *Q* values

(19) $$M_{q}\left(b\right)=<M_{q},b>=b_{q}$$

where $M_{q}$ are linear functionals to be selected. For the case of interest here, the linear functionals $M_{q}$’s correspond to elementary probes and their selection amounts at the determination of the positions of such elementary sensors.

By exploiting the available a priori information, *a* can be typically represented by means of its projection on a finite dimensional sub-space expanded by *N* basis functions $\psi_{n}$

(20) $$a=\sum_{n=1}^{N}a_{n}\psi_{n}.$$

The relation in Equation (18) is then discretized as

(21) $$\underline{b}=\underline{\underline{T}}\cdot \underline{a}$$

where $\underline{\underline{T}}$ is the matrix discretizing T, $\underline{a}=\left(a_{1},\ldots ,a_{N}\right)$ and $\underline{b}=\left(b_{1},\ldots ,b_{Q}\right)$.

The SVO consists into the determination of the parameters $\underline{p}$ and the set of the functionals $M_{q}$’s that improve the spectral behavior of the discrete counterpart $\underline{\underline{T}}$ of T [1,2,17].

The SVO is implemented by optimizing a proper Quality Factor Ξ expressed as a function of the Singular Values (SVs) of $\underline{\underline{T}}$. Ξ is strictly related to the amount of information carried by the data on the unknown. Although different possible choices are possible [43], in this paper Ξ is chosen to improve the Shannon number.

After the SVO, a regularized inversion is typically required. Indeed, with the SVO, not all the SVs can be retained “acceptable”. In this case, a partial reconstruction of the quantity of interest is performed.

Concerning now how many receivers should be allocated on the receiving region, *Q* is determined according to the iterative procedure illustrated in Algorithm 1. The saturation behavior is expected since adding further receivers does not increase the collectable amount of information which can be extracted from $A_{R}$ and needed to determine the characteristics of the transmitted field.
**Algorithm 1** SVO algorithm: determining the number of sensors.**Set**Q=Qinit≥N**1. Optimize**Ξ**to obtain**$\Xi_{opt}\left(Q\right)\;\;$**If the curve**$\Xi_{opt}\left(Q\right)$**is not saturated.**       Q=Q+1**goto** 1 **Choose**$Q_{opt}$**as the value of***Q***associated to the knee**

In the 2D case dealt with in this paper, the operator T coincides with the radiation operator A, *a* with the aperture field $E_{a}$, *N* in Equation (20) with $K_{T}$, the $\psi_{n}$’s in (20) with the $\Phi_{k}\left[c_{T};x^{\prime }\right]$’s and the $a_{n}$’s with the $e_{k}$’s. Moreover, the linear functionals $M_{q}$ are sampling functionals extracting the samples of the impinging field $E_{i}$. Accordingly, the elements of $\underline{b}$ are just the samples of $E_{i}$ and the parameters $\underline{p}$ coincide with the probe locations.

It should be noticed that, if the optimization parameters correspond to the spatial coordinates of the receivers, then *P* can be very large and this can affect an effective and efficient optimization. Therefore, to control the number of optimization parameters *P* defining the elementary probes, the non-uniform (x,y) grid is obtained by distorting a regular auxiliary grid (ξ,η) via a mapping function *r* to be determined, so that the *m*-th position is expressed as $\left(x^{\left(m\right)},y^{\left(m\right)}\right)=r\left(\xi^{\left(m\right)},\eta^{\left(m\right)}\right)$. The function *r* is represented by few, *P* basis functions $\tau_{s}$, namely,

(22) $$r\left(\xi ,\eta \right)=\sum_{s=1}^{P}p_{s}\tau_{s}\left(\xi ,\eta \right).$$

The approach can be considered as a pre-filtering/pre-conditioning strategy that reduces the degree of ill-conditioning of the relevant operator to be inverted.

### 4.3. Extended Elements

Finally, as an alternative to point-like elements, it is possible to use extended elements or even to mix point-like elements with extended elements. How the segmentation performs in solving the overlap issue between the “optimal virtual” sensors will be clearer in Section 5.4.

## 5. Performance Analysis and Discussion

In this section, we provide numerical results illustrating the performance of the introduced generalized Gaussian quadrature and the optimality of SVO. Unless explicitly mentioned, we will refer to a test case with $a_{T}=5\lambda$, $a_{R}=7\lambda$ and d=7λ.

We will first deal with the “optimal virtual” sensors benchmark and the problem of capturing all the radiated DoFs. Afterwards, we will analyze the performance of the generalized Gaussian quadrature, of SVO and of the partitioning approach.

### 5.1. “Optimal Virtual” Sensors and the Problem of Capturing all the Radiated DoFs

**Figure 7:**
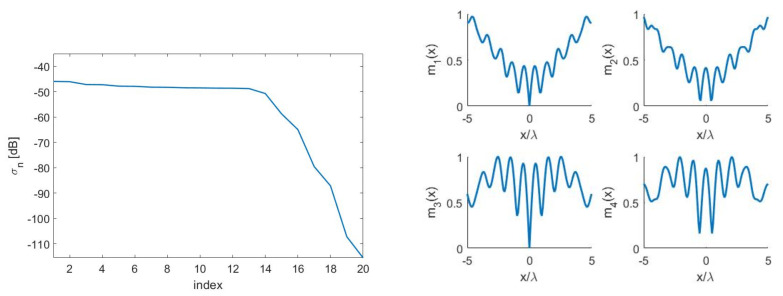
(**Left**): SVs for the case $a_{T}=5\lambda$, $a_{R}=7\lambda$ and d=7λ. (**Right**): first four “optimal virtual” sensors for the case $a_{T}=5\lambda$, $a_{R}=7\lambda$ and d=7λ.

Figure 7—left shows the (not normalized) SVs for the “optimal virtual” sensors case. Around $N_{opt}=16$ “optimal virtual” sensors were available corresponding to the SVs dropping by no more than 20dB as compared to the first one. Furthermore, in Figure 7—right, the first four “optimal virtual” sensing functions are displayed.

**Figure 8:**
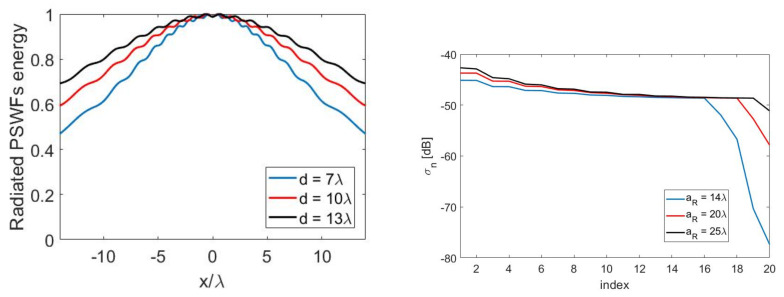
(**Left**): illustrating the spreading of the energy of the PSWFs for the case $a_{T}=5\lambda$ and $a_{R}=7\lambda$ and for an increasing distance *d*. (**Right**): increasing the size of $a_{R}$ to catch all the radiated DoFs for the case $a_{T}=5\lambda$ and d=10λ.

The spread of the energy of the radiated PSWFs with an increasing distance between transmitter and sensor is shown in Figure 8—left. Furthermore, for fixed values of $a_{T}=5\lambda$ and d=10λ, Figure 8—right shows how increasing $a_{R}$ to $a_{R}=25\lambda$ enabled us to recover all the $K_{T}=20$ radiated DoFs.

### 5.2. Gaussian Quadrature

**Figure 9:**
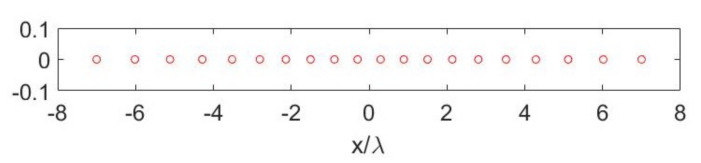
Optimized Gaussian quadrature points for the case $a_{T}=5\lambda$, $a_{R}=7\lambda$, d=7λ and N=20.

**Figure 10:**
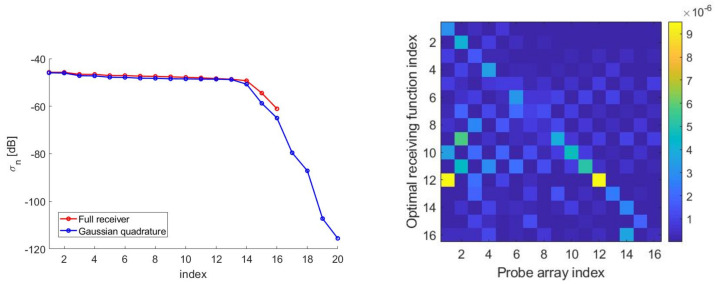
(**Left**): SVB for the case when “optimal virtual” sensing functions are used, instead of the PSWFs, to form the link matrix and the SVB obtained for the case of point-like elements and use of the “optimal” weights. (**Right**): percentage errors when the point-like elements when the “optimal” weights are used to form the elements of the link matrix instead of the “optimal virtual” sensors. The rows span the different sets of weights while the columns the possible impinging fields.

Figure 9 depicts the optimized Gaussian quadrature points for N=20 which was chosen to guarantee a maximum difference between left- and right-hand sides of Equation (17) less than $10^{-5}$. The number of elements was slightly larger than the number of $N_{opt}=16$ “optimal virtual” sensors to represent, as expected. The required number of elements was consistent with the results in [44]. Thanks to Figure 10—left, it was possible to compare the SVs for the case when “optimal virtual” sensing functions were used and the SVs obtained for the case of weighted point-like elements. As it can be seen, the two SVBs almost completely overlapped. Finally, Figure 10—right shows the differences between the left-hand side and the right-hand side of Equation (17) when “optimal” weights and positions were used. As can be seen, the signals sensed by the elementary arrays were very close to those sensed by the “optimal virtual” sensors. This is due to the capability of quadrature nodes and weights to represent the scalar products between the functions $m_{n}^{*}\left(x\right)$ and $A\left[\Phi_{k}\left[c_{T};x^{\prime }\right]\right]\left(x\right)$.

### 5.3. Elementary Sensors Using SVO

**Figure 11:**
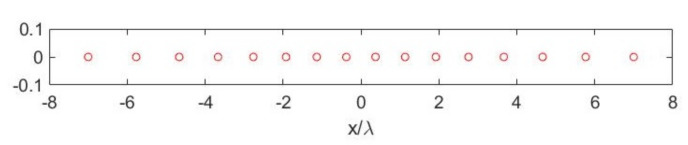
SVO points for the case $a_{T}=5\lambda$, $a_{R}=7\lambda$, d=7λ and N=20.

**Figure 12:**
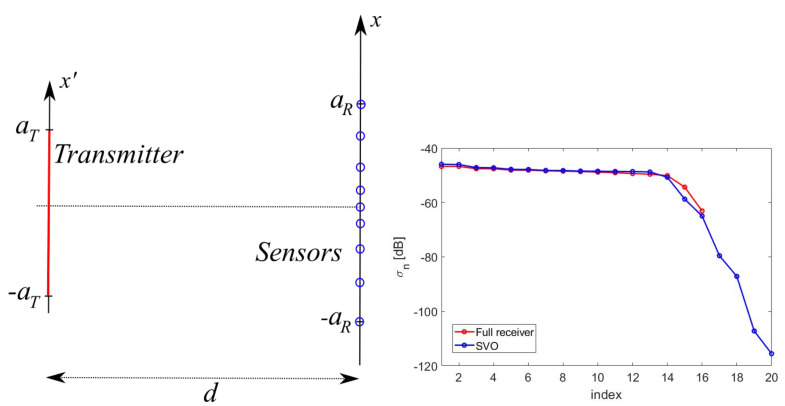
(**Left**): Illustrating the point-like sensor geometry without weights. (**Right**): SVO points for the case $a_{T}=5\lambda$, $a_{R}=7\lambda$, d=7λ and N=20.

Figure 11 depicts the optimized SVO points for the case illustrated in Figure 12—left. Moreover, Figure 12—right depicts the comparison between the SVs for the SVO points and for the “optimal virtual” sensors thus showing the optimality of SVO. As it can be seen, the SVBs were the same, indicating that the elementary optimized SVO elements had the same reconstruction potentialities of the extended “optimal virtual” sensors. Indeed, optimality was reached when the discretized operators obtained the same singular values dynamics of the continuous operators. Moreover, the number of array elements per probe as well as the reconstruction capabilities were essentially the same as for the generalized Gaussian quadrature case.

### 5.4. Partitioning the “Optimal Virtual” Sensors

We here considered the alternative solution to both the realization of the “optimal virtual” sensors and to the arrays of elementary sensors. We considered an intermediate solution by partitioning the sensing aperture to mitigate the overlap issue.

**Figure 13:**
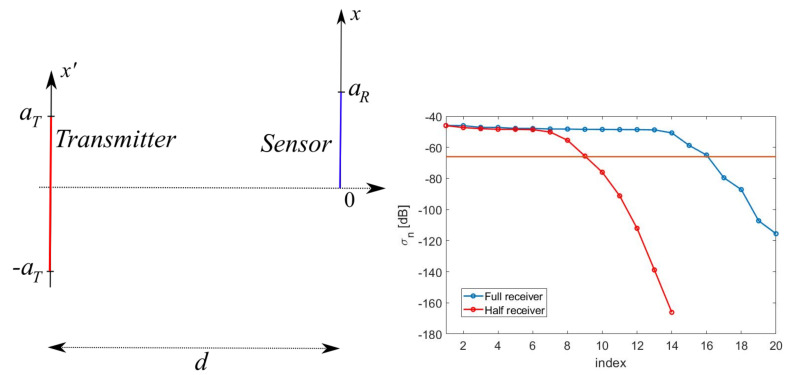
(**Left**): Illustrating the “optimal” reception problem for a halved upper aperture. (**Right**): SVB for the half-sensing aperture case.

**Figure 14:**
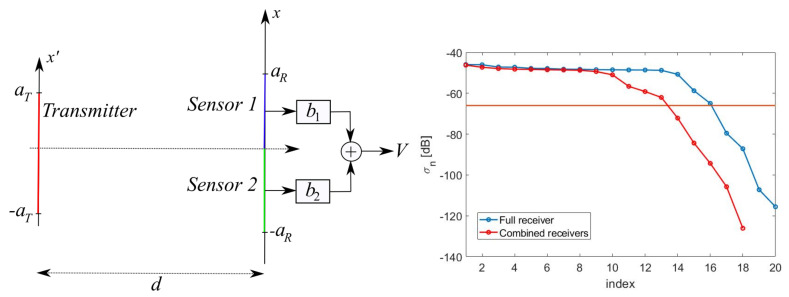
(**Left**): combining the “optimal” half-sensors with weights. (**Right**): SVB for the two half-sensors apertures combined with weights.

We first halved the aperture and deal with the upper half $\left(0,a_{R}\right)$ as in Figure 13—left. Figure 13—right shows the SVs as compared to those of the full aperture: the number of “optimal virtual” sensors was $N_{opt}^{\left(1\right)}=9$. Analogous results were achieved using $\left(-a_{R},0\right)$. This shows that, dealing with halved sensors, we had 18 “optimal virtual” sensors, 9 of them overlapping on the upper aperture and 9 of them overlapping on the lower aperture. At this point, we could combine the signal sensed by the two groups of 9 “optimal virtual” sensors using proper NWs $b_{1}$ and $b_{2}$ as in Figure 14—left. The NWs could be determined following the evaluation of the SVD of the link matrix whose generic element was evaluated as

(23) $$A_{kl}=\left\{\begin{array}{c} \langle A\left[\Phi_{k}\left[c_{T};x^{\prime }\right]\right]\left(x\right),\Psi_{l}^{\left(1\right)}\left(x\right)\rangle ,l\leq N_{opt}^{\left(1\right)}\\ \langle A\left[\Phi_{k}\left[c_{T};x^{\prime }\right]\right]\left(x\right),\Psi_{l}^{\left(2\right)}\left(x\right)\rangle ,N_{opt}^{\left(1\right)}+1\leq l\leq N_{opt}^{\left(1\right)}+N_{opt}^{\left(2\right)} \end{array}\right.,$$

where $\Psi_{l}^{\left(1\right)}\left(x\right)$ and $\Psi_{l}^{\left(2\right)}\left(x\right)$ are the “optimal virtual” sensing functions for the upper and lower half-apertures, respectively, and $N_{opt}^{\left(2\right)}=N_{opt}^{\left(1\right)}$. Figure 14—right shows the SVs for the above defined link matrix. As it can be seen, only a number of $13<N_{opt}=16$ independent NW combinations of the two groups of 9 “optimal virtual” sensors were obtained, meaning that we lost degrees of freedom.

Obviously, a further segmentation, although going in the direction of elementary sensors, would inherit the same efficiency problem associated to the sub-aperture recombination as before.

### 5.5. 3D Application: Far-Field Calculation from Near-Field Data

We finally present an application of the SVO to a 3D case consisting of the calculation of the field radiated by an aperture in the far-zone from near-field measurements.

We considered the case of a pyramidal horn antenna with physical aperture 3.018λ×2.348λ sized. The flare had a base width of 0.711λ×0.356λ and a height of 2.524λ and operates at 1.645 GHz. All the synthetic data were evaluated by FEKO and corrupted with noise having a Signal to Noise Ratio (SNR) of 35 dB.

**Figure 15:**
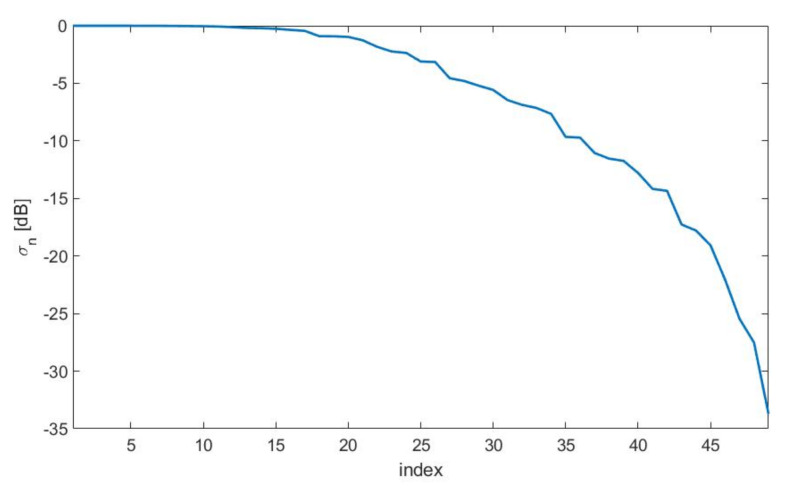
SVs for the horn antenna case.

**Figure 16:**
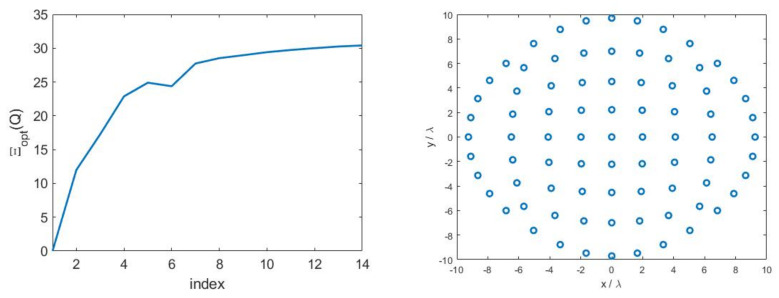
(**Left**): Curve of the optimized SVO functional with varying number of sampling points $\Xi_{opt}\left(Q\right)$. (**Right**): SVO sampling points.

For this test case, we considered an electrical aperture size of $2a_{T}\times 2b_{T}=3.5\lambda \times 3.05\lambda$ and a distance between aperture and measurement plane of d=7λ. Concerning the measurement plane, we chose $2a_{R}\times 2b_{R}=22\lambda \times 22\lambda$ since such a size brought all the SVs above a threshold set −35 dB below the maximum one, see Figure 15. In this way, we expected to recover all the source’s DoFs when the SNR was larger than 35 dB. On the other side, Figure 16—left shows the curve of the optimized SVO functional with varying number of sampling points $\Xi_{opt}\left(Q\right)$ where a distorted grid of Q×Q samples has been considered. As it can be seen, a number of Q=9 points along each dimension, leading to an overall number of 9×9 sampling points, was necessary to reach saturation [2]. Figure 16—right shows the sampling points produced by the SVO procedure.

**Figure 17:**
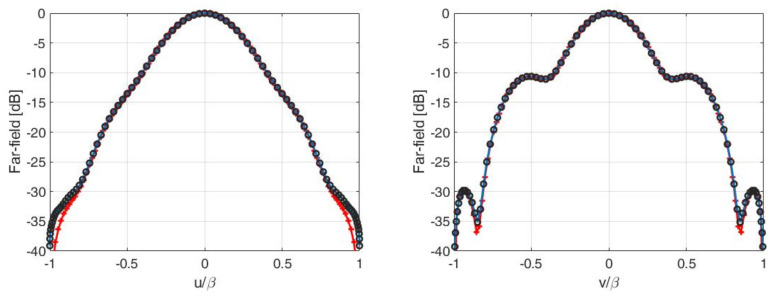
Cuts, along the *u*-axis (**left**) and *v*-axis (**right**), of the reference (red pluses) and reconstructed far-field using “optimal virtual” sensors (black circles) and SVO (blue solid line).

Figure 17 shows the cuts, along the *u* and *v* axes, of the reference far-field, as evaluated by FEKO, and that retrieved following the NFFF reconstruction using the “optimal virtual” sensors and SVO. The two reconstructions practically coincided.

In all the dealt-with cases, the difference between the reference curves and the reconstructions was due to the presence of the noise.

## 6. Conclusions

We have introduced the concept of “optimal virtual” receivers and we have shown that such receivers can be equivalently represented by an array. This can be achieved by two different approaches using a generalized Gaussian quadrature and SVO which lead to essentially the same results. We have also shown that an approach based on the use of subapertures is suboptimal.

The singular values have been exploited as a measure of the performance of the sensing and as the foundation for the comparisons. The singular vectors are used to design the link (“virtual” sensors).

The discussion has been led in a 2D, scalar setting. The theoretical arguments have been supported by numerical results referring to a Near-Field/Far-Field (NFFF) transformation problem.

The test reported throughout the paper and, in particular, the NFFF case, show that SVO is capable to reach the same results of the “optimal virtual” sensors so that it is “optimal” in turn. The capability of SVO to reaching the same SVB as for the “optimal virtual” sensors entails having an operator with the same performance in terms of conditioning and robustness against noise. However, thanks to its optimality, SVO involves the minimum number of field samples reaching the same performance of the continuous problem.

In the future, the case of arrays with parasitic elements can be also dealt with.

## Data Availability

Samples of the compounds are available from the authors.
